# Effect of PCSK9 inhibition in combination with statin therapy on intracranial atherosclerotic stenosis: A high-resolution MRI study

**DOI:** 10.3389/fnagi.2023.1127534

**Published:** 2023-03-09

**Authors:** Lingshan Wu, Qianqian Kong, Hao Huang, Shabei Xu, Wensheng Qu, Ping Zhang, Zhiyuan Yu, Xiang Luo

**Affiliations:** ^1^Department of Neurology, Tongji Hospital, Tongji Medical College, Huazhong University of Science and Technology, Wuhan, Hubei, China; ^2^Hubei Key Laboratory of Neural Injury and Functional Reconstruction, Huazhong University of Science and Technology, Wuhan, China

**Keywords:** PCSK9 inhibitor, statin, intracranial atherosclerosis, treatment, high-resolution MRI (HR-MRI)

## Abstract

**Introduction:**

Intracranial atherosclerotic stenosis (ICAS) is a common cause of stroke worldwide. Evolocumab, a proprotein convertase subtilisin/kexin type-9 inhibitor (PCSK9i), effectively lowers low-density lipoprotein (LDL) and produces favorable changes in coronary atherosclerosis. This study aimed to determine the effects of PCSK9i on intracranial plaques in moderate-intensity statin-treated individuals with ICAS.

**Methods:**

This prospective, observational study monitored the imaging and clinical outcomes of individuals with ICAS who were consecutively treated with moderate-intensity statins with or without PCSK9i. Individuals underwent monthly visits and repeat high-resolution MRI (HR-MRI) at week 12. The primary outcome was a change in HR-MRI after 12 weeks of treatment and the secondary outcome was major vascular events during follow-up.

**Results:**

Forty-nine individuals were studied (PCSK9i group: 26 individuals with 28 abnormal vascular regions; statin group: 23 with 27 regions). The PCSK9i group showed a significant reduction in the normalized wall index (0.83 vs. 0.86, *p* = 0.028) and stenosis degree (65.5 vs. 74.2%, *p* = 0.01). Similarly, a greater percentage of individuals with a good response to the efficacy of treatment were treated in the PCSK9i group than that in the statin group (75 vs. 44.4%, *p* = 0.021). The incidence of major vascular events was overall similar between the groups. The treatment options (OR = 8.441, *p* = 0.01) and prior diabetes (OR = 0.061, *p* = 0.001) were significantly associated with the efficacy of treatment.

**Discussion:**

Statin and PCSK9i combination treatment stabilized intracranial atherosclerotic plaques more often compared to statins alone, as documented by HR-MRI. Further study is warranted to determine if combination treatment improves clinical outcomes in ICAS.

## Introduction

Intracranial atherosclerotic stenosis (ICAS) is one of the most common causes of cerebrovascular ischemic events worldwide and is associated with stroke recurrence and higher mortality (Wang et al., 2014; Hurford et al., 2020; Gutierrez et al., 2022). The underlying mechanisms by which ICAS causes stroke include artery-to-artery embolism or arterial occlusion due to plaque rupture with *in situ* thrombosis, hemodynamic impairment due to highly stenotic plaques, and local small arterial branch origin occlusion (Qureshi and Caplan, 2014; Krasteva et al., 2020; Gutierrez et al., 2022). In the treatment of ICAS, data suggest that percutaneous transluminal angioplasty and stenting (PTAS) are inferior to medical therapy (Wang et al., 2020; Hurford and Rothwell, 2021). In the SAMMPRIS trial, a higher 12-month risk of stroke was reported in patients with ICAS who underwent PTAS plus aggressive medical management versus medical management alone. The SAMMPRIS trial was terminated early owing to a high rate of 30-day stroke or death in the PTAS group (Chimowitz et al., 2011). In the recent CASSISS trials, compared with medical therapy alone, the addition of PTAS to medical therapy in individuals with symptomatic ICAS resulted in no significant difference in the risk of stroke or death within 30 days or stroke in the qualifying artery beyond 30 days through 1 year (Gao et al., 2022).

Lipid-lowering treatment is an important part of the medical therapy of ICAS. Statins that work through inhibiting 3-hydroxy3-methylglutaryl coenzyme A reductase, stabilize arterial plaque in ICAS at high doses by lowering the serum low-density lipoprotein (LDL; Chung et al., 2020). Of importance, a 10% reduction in LDL was estimated to reduce the carotid intima-media thickness by 0.73% per year. Carotid intima-media thickness is a strong predictor of the development of carotid plaque and stenosis (Amarenco et al., 2004). However, high-dose statins are associated with side effects, such as muscle pain, abnormal liver dysfunction, and renal insufficiency. Further, there appears to be a dose response relationship between Atorvastatin and decreased liver function (Cai et al., 2021).

Proprotein convertase subtilisin/kexin type-9 inhibitor (PCSK9i) lower LDL levels by approximately 60% (Sabatine et al., 2017; Schwartz et al., 2018). PCSK9i decreased the lipid component and produced favorable anatomic changes in coronary atherosclerosis consistent with plaque stabilization and regression (Nicholls et al., 2022; Ota et al., 2022; Raber et al., 2022). However, the effects of PCSK9i on ICAS remain to be determined.

The STAMINA-MRI trial employed serial high-resolution MRI (HR-MRI) to assess the effect of statin treatment on ICAS. The study results indicated that high-dose statin treatment stabilized symptomatic intracranial atherosclerotic plaques (Chung et al., 2020). However, owing to more adverse effects, high-dose statins are impractical in practice, as confirmed in a recent study that moderate dose statin monotherapy predominated as a mode of lipid-lowering therapy (Ray et al., 2021). We conducted a prospective observational study to determine the effects of PCSK9i on ICAS in moderate-intensity statin-treated individuals.

## Materials and methods

### Study design

This prospective observational study evaluated individuals with ICAS who were consecutively treated with statins with or without PCSK9i from January 2021 to July 2022. The inclusion criteria were: (a) at least 18 years of age; (b) individuals who had symptomatic or asymptomatic ICAS (>50%), confirmed by computed tomographic angiography (CTA), magnetic resonance angiography (MRA), or digital subtraction angiography (DSA), at the proximal portion of the middle cerebral artery (MCA), basilar artery, or the intracranial portion of the internal carotid artery and vertebral artery; and (c) individuals who agreed to be enrolled in the trial and gave signed written informed consent. The exclusion criteria were: (a) individuals with extracranial artery stenosis >50%; (b) a history of moyamoya disease, vasculitis, arterial dissection, or etiologies other than atherosclerosis; (c) individuals allergic to alirocumab, atorvastatin, or gadolinium; (d) prior or current use of alirocumab; (e) individuals treated with other lipid-lowering drugs or combinations of such drugs; (f) individuals with severe hepatic or renal dysfunction; (g) inability to tolerate MRI or with poor HR-MRI imaging quality; and (h) individuals who lacked baseline HR-MRI or had incomplete follow-up data.

Study participants were divided into two groups according to their lipid-lowering drugs: the PCSK9i group and the statin group. Individuals who received Atorvastatin 10–40 mg/day were assigned to the statin group, while those who received both Atorvastatin at 10–40 mg/day and evolocumab at 140 mg every 2 weeks were assigned to the PCSK9i group. During treatment, the evolocumab dosage was without adjustment, while a reduced dose of statins was allowed if adverse effects were observed, including myalgia, elevated liver enzymes, or other side effects linked to statin therapy. During the treatment period, individuals underwent clinical (on-site) visits at weeks 4, 8, and 12, and repeated HR-MRI imaging at week 12. Laboratory studies were obtained before and during the 12-week treatment interval.

This study was approved by the ethics board of Tongji Hospital (Wuhan, China; No. TJ-IRB20210107). All study participants provided written informed consent.

### Clinical data

Clinical data including demographic information, vascular risk factors, and laboratory metrics were collected. Vascular risk factors included hypertension, diabetes, previous ischemic stroke, coronary artery disease, and smoking. The following laboratory data at admission and follow-up were collected: total cholesterol, triglyceride, high-density lipoprotein (HDL), and LDL.

### HR-MRI protocol

All the subjects underwent a 3.0 T MRI scanner (United Imaging Healthcare, Shanghai) with a 32-channel head coil. The imaging sequences and parameters were as follows: three-dimensional (3D) time-of-flight (TOF) MR angiography: repetition time (TR)/echo time (TE) of 18.6/3.4 ms, field of view (FOV) of 27.2 cm × 22 cm, flip angle of 16, slice thickness of 0.35, and 336 slices; diffuse-weighted imaging (DWI): repetition time (TR)/echo time (TE) of 4,930/99.20 ms, field of view (FOV) of 27.2 cm × 22 cm, flip angle of 90, slice thickness of 5, and 20 slices; precontrast and postcontrast T1-weighted (T1W): repetition time (TR) /echo time (TE) of 750/22.4 ms, field of view (FOV) of 27.2 cm × 22 cm, flip angle of 65, slice thickness 0.66, and 220 slices. Postcontrast images were acquired after intravenous injection of a contrast agent (Gadobenate Dimeglumine Injection; BRACCO, China; 0.2 mg/kg body weight).

### MRI imaging analysis

**Figure 1:**
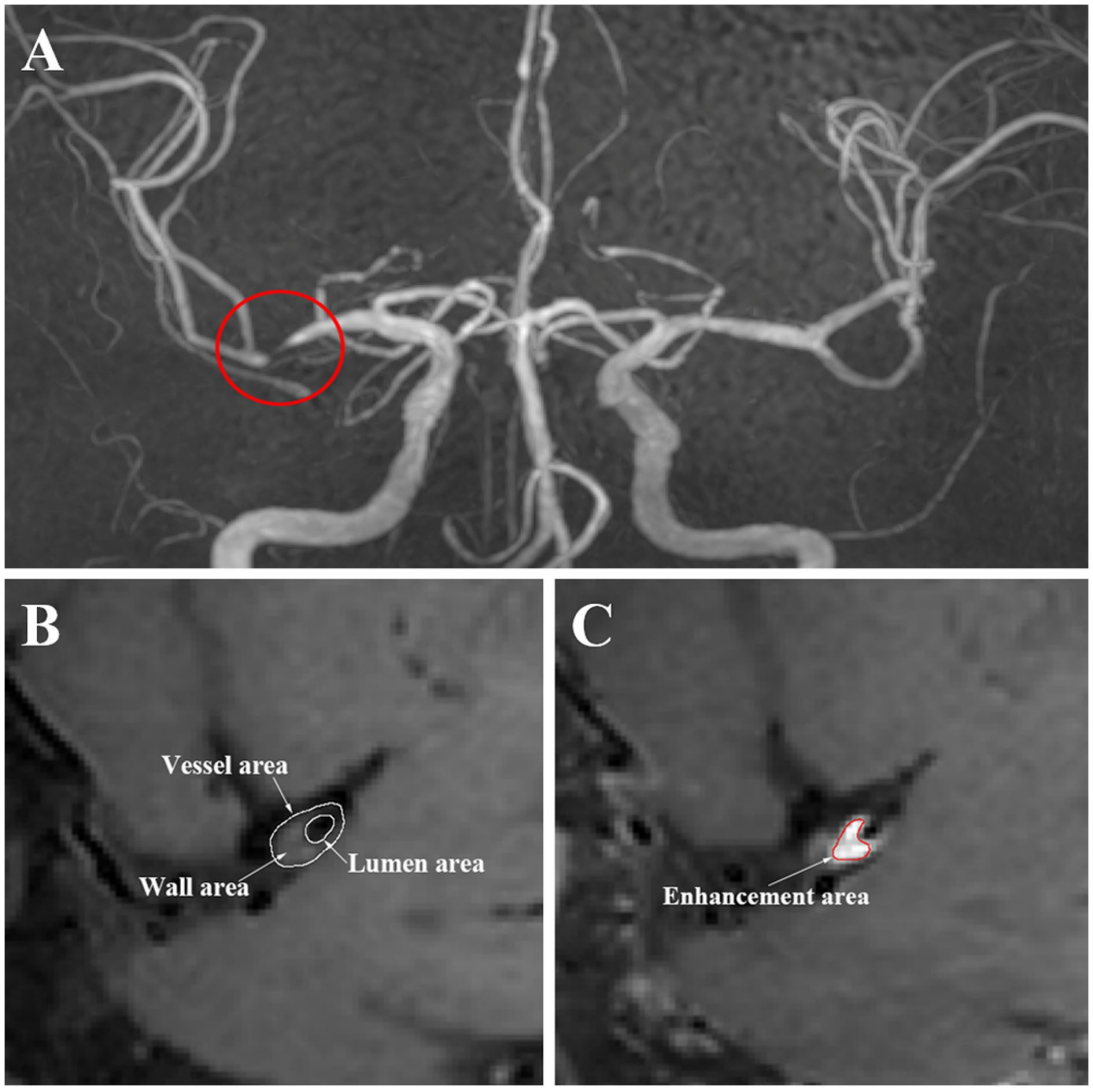
Illustration of HR-MRI arterial measurements.

The Medical Image Processing, Analysis, and Visualization (MIPAV) application was used to manually segment and extracted the characteristic of regions. First, based on the luminal image with TOF MR angiography, we selected the location of the most narrowed lumen (MNL) on the cross section. Second, we selected the reference site, which is the normal vessels located contralateral or proximal to the stenotic portion. Then, the vessel area (VA) and lumen area (LA) of the MNL and reference site were automatically calculated by the application after being traced and sketched manually. The wall area (WA) = VA−LA. The degree of stenosis was calculated as the stenosis degree = (1-LA_MNL_/LA_reference_) × 100% (Chung et al., 2020). The wall area index = WA_MNL_/WA_reference_. The normalized wall index = WA_MNL_/(LA_MNL_ + WA_MNL_) (Harteveld et al., 2018), which was used to evaluate the plaque burden (Sahota et al., 2021; Xiao et al., 2021). The remodeling index (RI) = VA_MNL_/VA_reference_. An RI ≥ 1.05 was defined as positive remodeling, 0.95–1.05 was intermediate remodeling, and an RI ≤ 0.95 was negative remodeling (Ma et al., 2010). The presence or absence and the pattern of enhancement were determined by the pre- and postcontrast T1 fluid-attenuated inversion recovery images. The presence of enhancement was defined as a > 20% increase in the normalized signal intensity of the plaque after contrast agent injection, which was calculated as signal intensity of the plaque/signal intensity of the mid pons. If the enhancement was uniform or circumferential, it was considered concentric; otherwise, it was regarded as eccentric. The enhanced area (EA) was manually drawn. The enhancement degree = EA_MNL_/WA_MNL_ × 100% (Ryoo et al., 2015). Figure 1 shows an illustration of HR-MRI measurements. The efficacy of treatment was defined as the change of stenosis degree. According to the percentage change of stenosis degree, individuals were categorized into two groups: good responders (<0) and poor responders (>0).

Evaluation of HR-MRI was conducted by two experienced observers (Lingshan Wu and Qianqian, Kong) who were blinded to treatment status and sequencing of imaging studies (baseline/follow-up). Disagreements were resolved by discussion, and when necessary, a third reader (Xiang Luo) with expertise in the field were consulted. The intraclass correlation coefficients for the measured HR-MRI parameters were above 0.80, which indicated good reliability.

### Outcomes

The primary endpoint was changes in HR-MRI variables before and after the 12-week treatment. Secondary endpoints were the major vascular events from baseline to 12 weeks, including vascular deaths, myocardial infarction (MI), and cerebrovascular events (ischemic stroke, transient ischemic attack, and hemorrhagic stroke).

### Adverse events

Any adverse events that occurred during the follow-up, such as muscle pain, liver dysfunction as determined by a change in laboratory data, allergy, and renal insufficiency, were reported.

### Statistical analysis

The statistical analysis was performed using SPSS version 26.0 (IBM, SPSS, Chicago, IL, United States). Continuous variables were compared using a *t*-test or the Mann–Whitney U-test. Categorical variables were compared with χ^2^ tests. Univariate logistic regression analysis was used to determine the factors associated with the efficacy of treatment. Variables with *p* < 0.1 in univariate analyses were included in the subsequent multivariable analysis using the forward stepwise method. Results were given by the odds ratio (OR) and 95% confidence interval (CI). A value of *p* < 0.05 was considered significant.

## Results

### Patient characteristic

**Figure 2:**
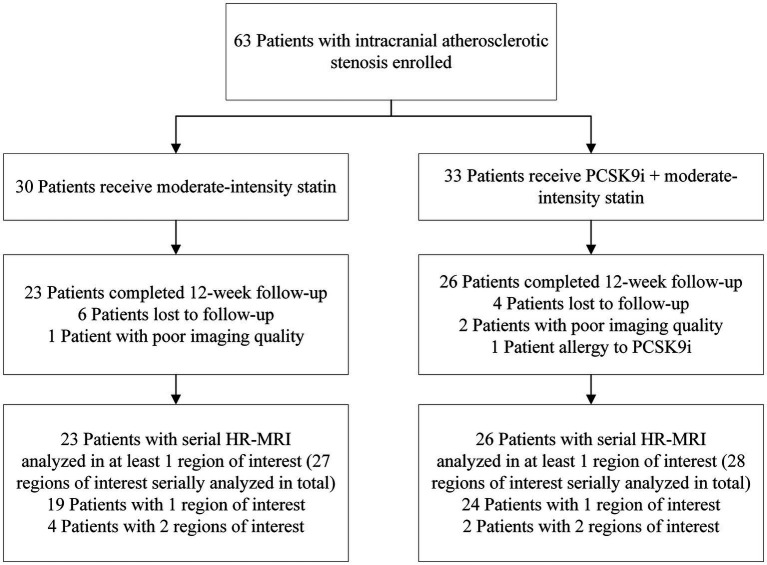
Cohort selection decision tree. PCSK9i, a proprotein convertase subtilisin/kexin type 9 inhibitor; HR-MRI, high-resolution MRI.

Of the 63 individuals evaluated, 49 with ICAS completed the 12-week treatment and were examined with follow-up HR-MRI (including 26 in the PCSK9i group and 23 in the statin only group). The disposition of individuals enrolled in the study is illustrated in Figure 2. Compared with the PCSK9i group, the statin group had a higher proportion of men (82.6 vs. 50%, *p* = 0.017). During the course of the study, the ratio of individuals who received 20 mg Atorvastatin between two groups was not different (82.6 vs. 92.3%, *p* = 0.400), nor was the ratio of those who received dual antiplatelet treatment (73.9 vs. 80.8%, *p* = 0.566). There was no significant difference in stroke risk factors, the remodeling pattern, or enhancement characteristics between the two groups. The clinical characteristics are summarized in Table 1.

**Table 1:** Baseline characteristics.

	Statin (*n* = 23)	PCSK9i (*n* = 26)	Value of *p*
Age (year), mean ± SD	50.96 ± 11.75	55.04 ± 12.81	0.253
Male sex, *n* (%)	19 (82.6)	13 (50)	**0.017**
Symptomatic, *n* (%)	14 (60.9)	21 (80.8)	0.124
Stroke risk factors, *n* (%)
Hypertension	18 (78.3)	15 (57.7)	0.125
Previous ischemic stroke	3 (13)	9 (34.6)	0.08
Diabetes	5 (21.7)	8 (30.8)	0.475
Coronary artery disease	0	2 (7.7)	0.491
Current smoking	14 (60.9)	9 (34.6)	0.066
Statin use, *n* (%)			0.400
Atorvastatin 40 mg	4 (17.4)	2 (7.7)	
Atorvastatin 20 mg	19 (82.6)	24 (92.3)	
Antiplatelet drug, *n* (%)			0.566
Dual	17 (73.9)	21 (80.8)	
Mono	6 (26.1)	5 (19.2)	
Target lesion, *n* (%)			0.118
Middle cerebral artery	11 (40.7)	17 (60.7)	
Internal carotid artery	2 (7.4)	1 (3.6)	
Basilar artery	3 (11.1)	6 (21.4)	
Vertebral artery	11 (40.7)	4 (14.3)	
Remodeling pattern, *n* (%)			0.578
Intermediate remodeling	0	1 (3.6)	
Negative remodeling	21 (77.8)	22 (78.6)	
Positive remodeling	6 (22.2)	5 (17.9)	
Enhancement in vessel, *n* (%)	18 (66.7)	22 (78.6)	0.322
Enhancement pattern, *n* (%)			0.360
Concentric	0	1 (4.5)	
Eccentric	18 (100)	21 (95.5)	

Value of *p*s < 0.05 are marked bold.

PCSK9i, a proprotein convertase subtilisin/kexin type 9 inhibitor.

### Laboratory data

There was no significant difference in laboratory results between the two groups at baseline (Table 2). Compared with the baseline, the total cholesterol (2.04 vs. 3.93, *p* < 0.001), triglycerides (0.94 vs. 1.37, *p* = 0.035), and LDL (0.64 vs. 2.38, p < 0.001) in the PCSK9i group were significantly decreased, while only the total cholesterol (2.81 vs. 3.36, *p* = 0.018) and LDL (1.44 vs. 2.03, *p* = 0.017) were significantly decreased in the statin group. Compared with the statin group, the total cholesterol (2.04 vs. 2.81, *p* < 0.001) and LDL (0.64 vs. 1.44, *p* < 0.001) on treatment in the PCSK9i group were significantly lower. The percentage change in the total cholesterol (−47.4 vs. −21.6%, *p* < 0.001) and LDL (−68.3 vs. −32.1%, *p* < 0.001) were significantly greater in the PCSK9i group than in the statin group.

**Table 2:** Laboratory data.

	Statin (*n* = 23)	PCSK9i (*n* = 26)	Value of *p*
Total cholesterol (mmol/L)
Baseline	3.36 (2.87 ~ 3.98)	3.93 (2.59 ~ 4.36)	0.385
On treatment	2.81 (2.33 ~ 3.78)	2.04 (1.79 ~ 2.21)	**<0.001**
Percent change	−21.6 (−34.1 ~ 3.2)	−47.4 (−57.2 ~ −24.6)	**<0.001**
*p* value from baseline	**0.018**	**<0.001**	
Triglyceride (mmol/L)
Baseline	1.53 (0.91 ~ 2.04)	1.37 (1.11 ~ 1.83)	0.930
On treatment	1.14 (0.85 ~ 1.53)	0.94 (0.79 ~ 1.49)	0.522
Percent change	−22.5 (−40.6 ~ 27.3)	−27.6 (−44.4 ~ −3.1)	0.272
Value of *p* from baseline	0.358	**0.035**	
HDL (mmol/L)
Baseline	0.99 ± 0.26	0.95 ± 0.19	0.650
On treatment	1.01 ± 0.22	1.06 ± 0.27	0.491
Percent change	2.3 ± 14.4	11.8 ± 24.1	0.137
Value of *p* from baseline	0.773	0.129	
LDL (mmol/L)
Baseline	2.03 (1.54 ~ 2.69)	2.38 (1.25 ~ 2.82)	0.644
On treatment	1.44 (1.20 ~ 1.94)	0.64 (0.43 ~ 0.76)	**<0.001**
Percent change	−32.1 (−42.7 ~ 4.2)	−68.3 (−81.3 ~ −52.6)	**<0.001**
Value of *p* from baseline	**0.017**	**<0.001**	

Value of *p*s < 0.05 are marked bold.

PCSK9i, a proprotein convertase subtilisin/kexin type 9 inhibitor; HDL, high-density lipoprotein; and LDL, low-density lipoprotein.

### Primary and secondary endpoints

There was no significant difference in HR-MRI findings between the two groups at baseline. The stenosis degree decreased from 74.2 to 65.5% in the PCSK9i group (*p* = 0.010 for comparison from baseline). A significant reduction in the normalized wall index (0.83 vs. 0.86, *p* = 0.028) was observed in the PCSK9i group but not the statin group. Similarly, a greater percentage of individuals with a good response to the efficacy of treatment were treated in the PCSK9i group than that in the statin group (75 vs. 44.4%, *p* = 0.021). However, the wall area index, remodeling index, enhancement area, and enhancement degree were not significantly different, regardless of PCSK9i administration. Details of the HR-MRI findings before and after treatment are summarized in Table 3.

**Table 3:** HR-MRI changes.

	Statin (number of regions = 27)	PCSK9i (number of regions = 28)	Value of *p*
Wall area index
Baseline	1.05 (0.90–1.53)	1.14 (0.77–1.45)	0.798
Follow-up at week 12	1.10 (0.83–1.67)	1.103 (0.83–1.44)	0.481
Percentage change	−1.2 (−7.68–11.6)	−4.9 (−18.1–11.3)	0.350
*p* value from baseline	0.883	0.635	
Normalized wall index
Baseline	0.83 ± 0.048	0.86 ± 0.06	0.062
Follow-up at week 12	0.84 ± 0.05	0.83 ± 0.06	0.496
Percentage change	1.1 ± 4.4	−3.8 ± 4.5	**<0.001**
*p* value from baseline	0.532	**0.028**	
Stenosis degree (%)
Baseline	69.3 ± 12.6	74.2 ± 10.4	0.141
Follow-up at week 12	71.0 ± 12.8	65.5 ± 13.8	0.103
Percentage change	3.2 ± 14.2	−12.0 ± 13.1	**<0.001**
*p* value from baseline	0.620	**0.010**	
The efficacy of treatment, *n* (%)			**0.021**
Good responder	12 (44.4)	21 (75)	
Poor responder	15 (55.6)	7 (25)	
Remodeling index
Baseline	0.72 (0.56–0.93)	0.73 (0.56–0.92)	0.566
Follow-up at week 12	0.73 (0.58–0.89)	0.79 (0.54–0.93)	0.768
Percentage change	−2.7 (−7.3 ~ 4.3)	0.31 (−11.5 ~ 13.7)	0.614
*p* value from baseline	0.849	0.787	
Enhancement area (EA)
Baseline	10.91 ± 7.82	8.63 ± 6.67	0.113
Follow-up at week 12	10.71 ± 7.41	8.01 ± 6.35	0.113
Percentage change	−1.2 ± 29.6	−8.3 ± 31.9	0.421
*p* value from baseline	0.929	0.722	
Enhancement degree
Baseline	0.61 ± 0.17	0.64 ± 0.21	0.711
Follow-up at week 12	0.58 ± 0.22	0.61 ± 0.31	0.710
Percentage change	−3.2 ± 29.5	−1.8 ± 39.7	0.894
*p* value from baseline	0.573	0.697	

Value of *p*s < 0.05 are marked bold.

PCSK9i, a proprotein convertase subtilisin/kexin type 9 inhibitor.

During the follow-up, one individual in each group experienced a major vascular event (Table 4).

**Table 4:** Major vascular events during follow-up.

Patient No.	Age	Sex	Site	Clinical presentation	Treatment	Outcome
1	43	Female	LMCA	symptomatic	PCSK9i + moderate-intensity statins	Within 12 weeks of follow-up, this patient experience TIA twice manifested by right hemiparesis.
2	34	Female	RICA	asymptomatic	moderate-intensity statins alone	The patient experienced a right hemispheric stroke 6 weeks after the baseline HR-MRI.

PCSK9i, a proprotein convertase subtilisin/kexin type 9 inhibitor MCA, middle cerebral artery; ICA, internal carotid artery; and TIA, transient ischemic attack.

### Safety

As shown in Table 5, the ratio of individuals with abnormal liver dysfunction was similar between two groups (4.3 vs. 7.7%, *p* = 1). During the follow-up, no other adverse events occurred.

**Table 5:** Adverse events.

Events	Statin (*n* = 23)	PCSK9i (*n* = 26)	Value of *p*
Muscle problems, *n* (%)	0	0	
ALT >3 × ULN, *n* (%)	1 (4.3)	2 (7.7)	1
Renal insufficiency, *n* (%)	0	0	
General allergic reaction, *n* (%)	-	0	
Local injection site reaction, *n* (%)	-	0	
Other, *n* (%)	0	0	

As shown in Figure 2, one patient had a general allergic reaction to PCSK9i and was excluded to this study.

PCSK9i, a proprotein convertase subtilisin/kexin type 9 inhibitor ALT, alanine aminotransferase; and ULN, upper limit of normal.

### Exploratory analyses

Based on these results, univariate and multiple logistic regression analyses were performed to identify the independent factors associated with the efficacy of treatment Among the clinical and HR-MRI variables, the treatment options (OR = 8.441, *p* = 0.01) and prior diabetes (OR = 0.061, *p* = 0.001) were significantly associated with the efficacy of treatment (Table 6).

**Table 6:** Univariate and multiple logistic regression analyses for the efficacy of treatment.

	Univariate analysis		Multivariate analysis		OR (95% CI)	Value of *p*	OR (95% CI)	Value of *p*
PCSK9i group	3.75 (1.195–11.768)	**0.023**	8.441 (1.668–42.708)	**0.01**
Age	0.993 (0.948–1.039)	0.749		
Male sex	0.75 (0.229–2.453)	0.634		
Hypertension	0.342 (0.094–1.241)	0.103		
Diabetes	0.124 (0.035–0.443)	**0.001**	0.061 (0.011–0.329)	**0.001**
Previous ischemic stroke	1.957 (0.526–7.276)	0.317		
Coronary artery disease	-	0.999		
Current smoking	1.062 (0.361–3.126)	0.912		
Enhancement	0.444 (0.121–1.636)	0.223		
Wall area index	2.042 (0.689–6.046)	0.198		
Normalized wall index	-	0.209		
Remodeling index	0.705 (0.305–1.629)	0.413		
Enhancement area (EA)	0.961 (0.891–1.035)	0.291		
Enhancement degree	0.054 (0.003–1.107)	0.058		

Value of *p*s < 0.05 are marked bold.

PCSK9i, a proprotein convertase subtilisin/kexin type 9 inhibitor.

## Discussion

This study assessed the effects of PCSK9i on intracranial plaques in moderate-intensity statin-treated individuals with ICAS. Our findings show that the addition of PCSK9i to statins, compared with statins alone, resulted in a greater reduction in both the LDL and stenosis degree after 12 weeks. Treatment with PCSK9i lowered the LDL from 2.38 to 0.64 mmol/L and the mean stenosis degree from 74.2 to 65.5%. The plaque burden also decreased than baseline, with NWI from 74.2 to 65.5%. Based on the exploratory analyses, our findings indicate that the addition of PCSK9i to statins is a favorable factor for the improvement of the stenosis degree, while diabetes is a risk factor.

Individuals with diabetes might have a higher risk of ICAS (Qureshi and Caplan, 2014; Ma et al., 2019b). We found that diabetes is a risk factor for the improvement of the stenosis degree. Diabetes is associated with increased plaque burden, healed plaque ruptures, and positive remodeling, along with greater calcification in type 2 diabetes (Yahagi et al., 2017). Inflammatory (macrophages and T lymphocytes) infiltrate and necrotic core is greater in diabetes vs. non-diabetics. Moreover, diabetes is associated with increased plaque burden, positive remodeling, and calcification (Yahagi et al., 2017).

Plaque rupture with *in situ* thrombosis is one of the most important causes of ICAS-associated stroke, which can produce artery-to-artery embolism or occlusion of the artery (Bentzon et al., 2014; Gutierrez et al., 2022). Data indicate that lower LDL levels are associated with regress of atherosclerosis (Nissen et al., 2006). Similarly lower LDL levels are associated with reduce the risk of atherosclerotic cardiovascular disease in individuals with coronary artery disease (Ference et al., 2017). The STAMINA-MRI trial showed that high-dose statin treatment stabilized symptomatic intracranial atherosclerotic plaques (Chung et al., 2020). The lowering of blood lipid levels is especially helpful for stroke risk reduction in individuals with large artery atherosclerosis plaque (Hindy et al., 2018). Compared with less-intensive lipid-lowering statin-based therapies, more intensive therapies may be associated with a reduced risk of recurrent stroke in individuals with atherosclerosis (Lee et al., 2022).

Latest guidelines suggest an LDL reduction of ≥50% from baseline and an LDL goal of <1.4 mmol/L for secondary prevention are desirable in very-high-risk individuals (Mach et al., 2020). Log-linear dose–response after statin treatment were observed, consistent with the so-called “rule of 6%,” which describes the additional percentage reduction in the LDL from pretreatment for each statin dose doubling (Oni-Orisan et al., 2018). While there was also a dose–response relationship between statin and liver dysfunction, this relationship was only determined in Atorvastatin (Cai et al., 2021). Given the side effects and dose–response effect of statin, the target LDL level is hard to achieve with a statin alone. LDL receptors on the hepatocyte cell membrane can decrease the levels of circulating LDL particles by binding to the LDL particles/LDL receptor complex (Gallego-Colon et al., 2020). At the lysosome, LDL particles are recycled into esterified cholesterol and triglycerides to carry various roles within the cell. Similarly, the LDL receptor can be recycled back to the cell surface (Gallego-Colon et al., 2020).

Proprotein convertase subtilisin/kexin type-9 inhibitor can diminish the clearance of LDL from circulation by increasing LDL receptors to catabolism in the hepatocyte and blocking the normal recycling of the LDL receptor to the surface of the hepatocyte (Cohen et al., 2006; Taylor and Thompson, 2016). PCSK9i is a monoclonal antibody that effectively lowers LDL levels by approximately 60% (Sabatine et al., 2017; Schwartz et al., 2018). Similarly, statins inhibit the rate-limiting step of cholesterol biosynthesis up-regulating hepatic LDL receptors expression (Nurmohamed et al., 2021). In the GLAGOV trial, individuals with coronary artery disease treated with statins and PCSK9i had a greater decrease in percentage atheroma compared with those given statins alone (Nicholls et al., 2016). The Fourier trial found that PCSK9i in a background of statin therapy lowered LDL levels to a median of 30 mg/dL and reduced the risk of cardiovascular events (Sabatine et al., 2017). We found that compared with moderate-intensity statins alone, the addition of PCSK9i significantly reduced the stenosis degree in individuals with ICAS, which suggests that PCSK9i can stabilize and regress intracranial atherosclerosis. Increased fibrous cap thickness and decreased macrophage accumulation grade were greater with PCSK9i and statin combination treatment than with the statin treatment alone. Matrix metalloproteinases released from the accumulated macrophages can degrade collagen tissue of the fibrous cap, which is a major determinant of plaque vulnerability (Yano et al., 2020). Similarly, the percentage change in lipid arc was greater in the statin plus PCSK9i group vs. the statin alone (Yano et al., 2020). Note that compared with baseline, the stenosis degree after treatment made no difference among individuals in statin group. This differs from the STAMINA-MRI trial. In the STAMINA-MRI trial, all the subjects included under the 6-month high-dose (40–80 mg Atorvastatin or 20 mg Rosuvastatin) statin treatment, with LDL decreased from 3.25 to 1.58 mmol/L. While in this study, 82.6% of patients received Atorvastatin 20 mg/day for 12 weeks in the statin group and the degree of LDL decrease is lower than in the STAMINA-MRI trial (from 2.03 to 1.44 mmol/L). As mentioned above, the greater the LDL reduction, the greater the carotid intima-media thickness reduction (Amarenco et al., 2004). Therefore, the difference between the two studies perhaps secondary to the lower statin dose and shorter follow-up time of our study. The incidence of major vascular events was similar overall between the groups during the short duration of our study. What is more, there were no hemorrhagic strokes observed in the PCSK9i group, despite LDL levels ranging from 2.38 to 0.64 mmol/L. This is relevant, as others noted that lower LDL levels were associated with a higher risk of hemorrhagic stroke (Wang et al., 2013; Ma et al., 2019a).

### Strengths and limitations

This study had several strengths. To the best of our knowledge, it was the first to evaluate the effects of PCSK9i on intracranial plaques in moderate-intensity statin-treated individuals with ICAS through HR-MRI. The study design permitted an assessment of the effect of PCSK9i on the background of moderate statin therapy. Another plus was the overall rigor of the cohort determination, such that only individuals with ICAS and atherosclerosis were eligible for inclusion.

Several limitations should be noted. First, this was a prospective but observational study. Inherent selection bias may have occurred among the groups. Second, the relatively small number of study subjects and the short interval between baseline and follow-up HR-MRI were other limitations of this study. Data indicate that 12-week-treatment of statin and PCSK9i combination treatment produced incremental growth in fibrous-cap thickness and regression of the lipid-rich plaque in coronary artery disease (Yano et al., 2020), while the short interval predicted to under emphasize vascular changes occurring beyond 12 weeks. Based on the present results, larger and longer-term studies should investigate PCSK9i in individuals with ICAS. Third, all analysis in this study was performed at a single level, the location of the most severe stenosis. Others applied 3D volumetric analysis to assess carotid plaque (Sadat et al., 2010). However, volumetric analysis could not be performed in the present study secondary to the small diameter of the intracranial arteries (Ryoo et al., 2015). Last, treatment with antiplatelet, antihypertensive, and hypoglycemic drugs may alter with the progression of atherosclerotic plaques and the major vascular events in individuals with ICAS.

In conclusion, this study evaluated the efficacy of PCSK9i on intracranial plaques in moderate-intensity statin-treated patients with ICAS. Addition of PCSK9i to moderate statin therapy, compared with statins alone, significantly reduced the stenosis degree. Further research is needed to understand whether this combination improves clinical outcomes in ICAS.

## Data availability statement

The raw data supporting the conclusions of this article will be made available by the authors, without undue reservation.

## Ethics statement

The studies involving human participants were reviewed and approved by Tongji Hospital, Tongji Medical College, Huazhong University of Science and Technology. The patients/participants provided their written informed consent to participate in this study.

## Author contributions

XL, SX, and WQ: concept and design. LW, QK, HH, and XL: acquisition of data. LW, HH, and PZ: statistical analysis. LW: drafting of the manuscript. LW, QK, HH, SX, WQ, PZ, ZY, and XL: critical revision of the manuscript for important intellectual content. All authors contributed to the article and approved the submitted version.

## Funding

This study was supported by the National Nature Science Foundation of China (82171385 to XL), Key Research and Development Program of Hubei Province (2020BCA070 to XL), the Application Foundation Frontier Special Project of Wuhan Science and Technology Bureau (2020020601012226 to XL), and the Flagship Program of Tongji Hospital (2019CR106 to XL).

## Conflict of interest

The authors declare that the research was conducted in the absence of any commercial or financial relationships that could be construed as a potential conflict of interest.

## Publisher’s note

All claims expressed in this article are solely those of the authors and do not necessarily represent those of their affiliated organizations, or those of the publisher, the editors and the reviewers. Any product that may be evaluated in this article, or claim that may be made by its manufacturer, is not guaranteed or endorsed by the publisher.
